# Supplementary material for: Myelin Quantification in White Matter Pathology of Progressive Multiple Sclerosis Post-Mortem Brain Samples: A New Approach for Quantifying Remyelination
Source: Int J Mol Sci. 2021 Nov 23;22(23):12634. doi: 10.3390/ijms222312634 (PMC8657470; doi:10.3390/ijms222312634)
Supplement: Supplementary file 1 [file ijms-22-12634-s001.zip › ijms-1455836-supplementary.pdf]

Table S1. Patient characteristics of MS patients and Non-neurological controls (NNCs).

Patient No.	Sex	Disease type	Age at death (y)	Age at diagnosis (y)	Disease duration (y)	Cause of death	PMD (hh:mm)	No. of tissue blocks	Lesions (N)	Average RM-%	% of lesions with RM	Mean myelin-% in NAWM[1]
1	M	PPMS	57	32	25	Sepsis, UTI	3:00	1	2	0	0%	93.545
2	F	UNK	61	59	2	Euthanasia	4:30	2	4	6.688	25%	97.351
3	F	PPMS	57	32	25	Euthanasia	4:00	5	7	53.769	85.7%	96.422
4	M	SPMS	82	38	44	Pneumonia	3:00	1	1	0	0%	93.381
5	M	SPMS	50	31	19	Euthanasia	5:30	2	2	18.067	100%	98.738
6	F	SPMS	82	22	60	Euthanasia	3:40	1	1	75.608	100%	97.744
7	F	SPMS	53	37	16	Euthanasia	4:00	4	3	52.241	100%	95.213
8	F	UNK	87	69	18	Dehydration	6:00	1	1	0	0%	98.384
9	F	UNK	48	UNK	UNK	Exhaustion	6:00	6	18	25.354	44.4%	96.951
10	F	PPMS	51	36	15	Euthanasia	4:15	2	4	35.555	75%	97.882
11	F	SPMS	35	25	10	Euthanasia	4:00	4	7	11.931	57.1%	97.948
12	M	SPMS	70	23	47	Cardiac failure, clostridium difficile colitis	5:30	1	3	0	0%	96.561
13	F	SPMS	60	38	22	Euthanasia	5:10	1	1	0	0%	98.588
14	M	SPMS	54	33	21	Euthanasia	4:15	1	1	19.950	100%	97.637
1 (NNC)	F		58			MODS	6:15	1				97.420
2 (NNC)	F		69			Cardiogenic shock	6:15	1				99.180
3 (NNC)	F		61			Cachexia	10:15	1				94.300
4 (NNC)	M		70			Pancreas carcinoma	7:30	1				95.190
5 (NNC)	M		62			Adeno-carcinoma	6:35	1				98.630
**Total MS**	35.7% Males	21.4% PPMS						32	55		55% (30/55)	
**Total NNC**	40% Males							5				
**Mean (SD) MS**			60.5 (14.26)	36.83 (13.422)	24.923 (15.479)			2.28 (1.666)	3.929 (4.383)	21.369 (23.547)	49% (0.414)	96.881 (1.713)
**Mean (SD) NNC**			64 (5.244)					1 (0.000)				96.944 (1.905)

Descriptives: y = years, h = hours, m = minutes, PMD = post mortem delay, RM-% = percentage remyelination, RM = remyelination, NAWM = Normal appearing white matter, NNC = Non-neurological control, SD = standard deviation, UNK = unknown. MODS = multiple organ dysfunction syndrome.
[1] In this column the percentage of myelin is given for the WM in MS patients in the NAWM and for the NNCs in the normal WM (NWM).

Table S2. Quality analysis of the myelin percentage computation in lesions with different NAWM magnifications

Lesion 1: demyelinated lesion (RM score 0)	Black pixel percentages ((P_B/P_T) x 100%)	Estimated percentage myelin within lesion (myelin-% in lesion ROI/average myelin-% in NAWM ROIs x 100%)
Analysis 1a[1] Different magnifications:		
Lesion: 4.0x	0.44% myelin	0.44%/(99.26+99.55+96.22/3) x 100%
NAWM region 1: 2.3x	99.26% myelin	
NAWM region 2: 4.0x	99.55% myelin	= **0.45%** myelin left within the lesion
NAWM region 3: 6.3x	96.22% myelin	
Analysis 1b[2] Equal magnifications:		
Lesion: 4.0x	0.44% myelin	0.44%/(95.80+99.55+96.77/3) x 100%
NAWM region 1: 4.0x	95.80% myelin	
NAWM region 2: 4.0x	99.55% myelin	= **0.45%** myelin left within the lesion
NAWM region 3: 4.0x	99.77% myelin	
**Lesion 2: remyelinated lesion (RM score 3)**	**Black pixel percentages** ((P_B/P_T) x 100%)	**Estimated percentage myelin within lesion** (myelin-% in lesion ROI/average myelin-% in NAWM ROIs x 100%)
Analysis 2a[1] Different magnifications:		
Lesion: 2.6x	49.68% myelin	49.68%/(98.63+97.77+98.48/3) x 100%
NAWM region 1: 1.7x	98.63% myelin	
NAWM region 2: 2.6x	97.77% myelin	= **50.54**% remyelination
NAWM region 3: 3.6x	98.48% myelin	
Analysis 2b[2] Equal magnifications:		
Lesion: 2.6x	49.68% myelin	49.68%/(97.42+97.77+98.30/3) x 100%
NAWM region 1: 2.6x	97.42% myelin	= **50.78**% remyelination
NAWM region 2: 2.6x	97.77% myelin	
NAWM region 3: 2.6x	98.30% myelin	

[1]Analysis **1a** and **2a** are exemplifying the computation of the myelin percentages in a demyelinated and remyelinated lesion, respectively, performed with three different magnifications of the NAWM regions. [2]Analysis **1b** and **2b** are showing the myelin percentage computation in the same lesions, but with the associated three NAWM regions in the same magnification as the lesion magnification. RM = remyelination, P_B = black pixel count, P_T = total pixel count, myelin-% = percentage myelin, ROI = region of interest, NAWM = normal appearing white matter.

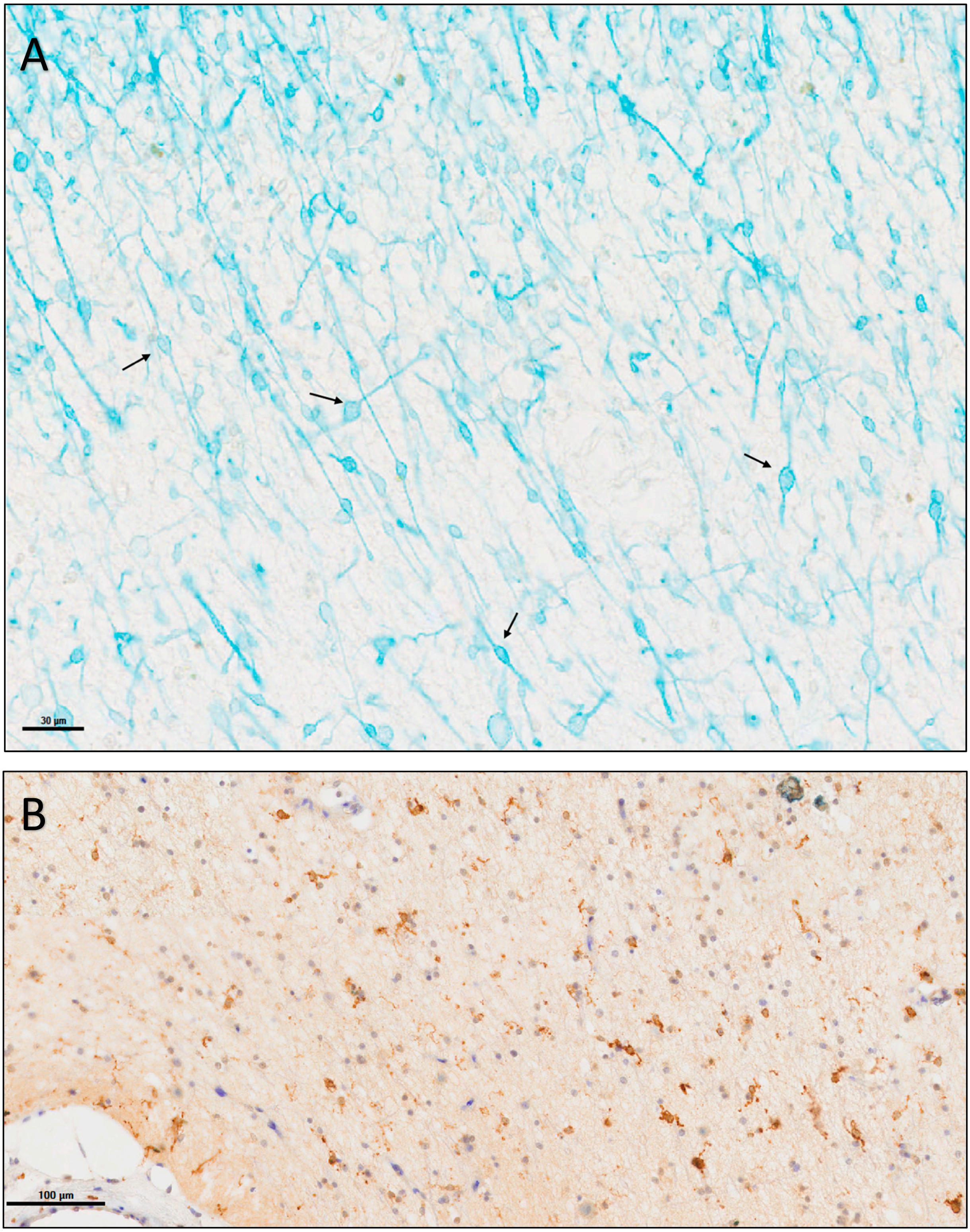

Figure S1. Picture of spheroid formation in myelin fibers and corresponding number of macrophages/microglia-cells. **(A)** Myelin spheroids are visible in myelin fibers of an active demyelinating WM lesion (LFB-stained section); **(B)** Represents the corresponding MHC-II-stained (LN3) brain region of figure A and shows activated microglia-cells. These microglia indicate that this WML is immune-activated, a so-called 'active WM lesion'.

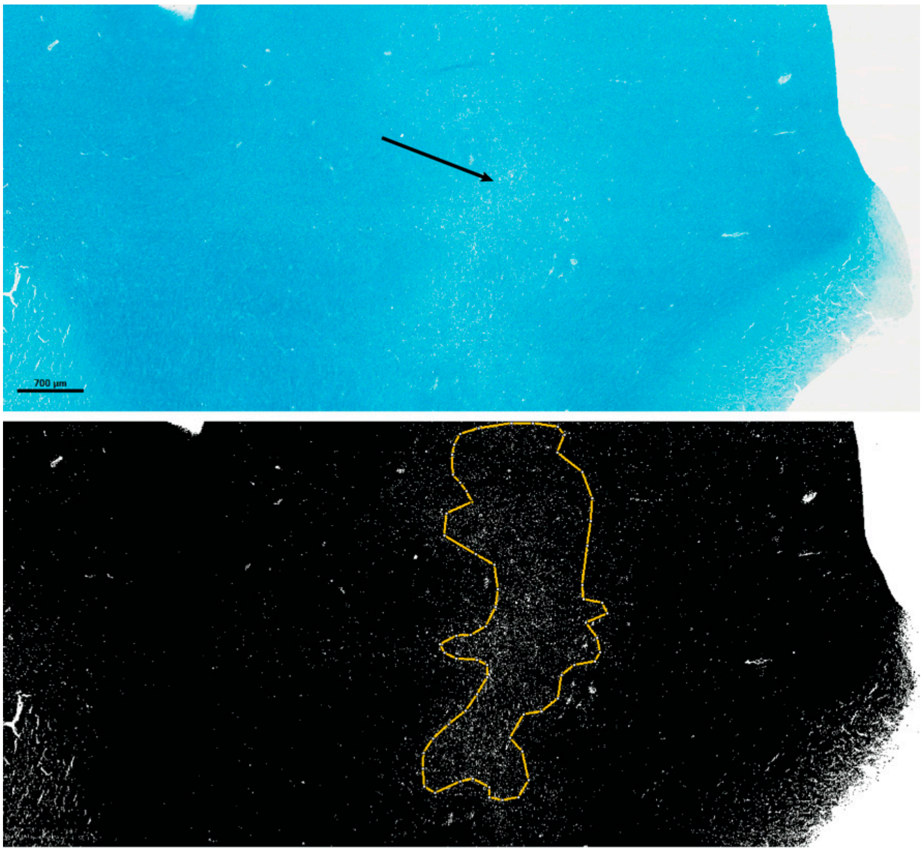

Figure S2. Thresholded image of DAWM. **(A)** Presents the LFB-stained image with a diffuse lighter area (DAWM) indicated by the arrow; **(B)** The black and white image and its ROI surrounding DAWM, with a threshold value of 201 and a myelin density of 93.11% after correction with NAWM. LFB = luxol fast blue, NAWM = normal appearing white matter, GM = grey matter, DAWM = diffusely appearing white matter, ROI = region of interest.

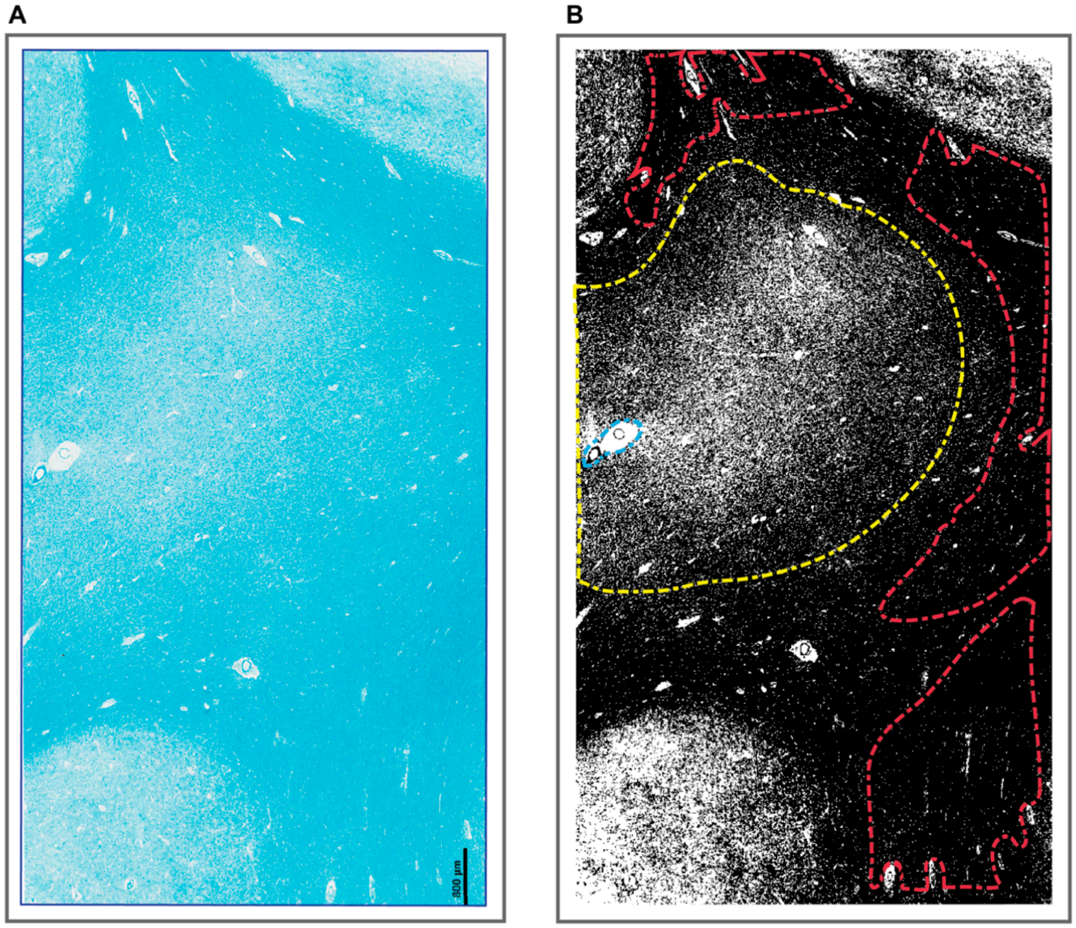

NAWM ROIs

	Black Pxls NAWM	Tot. Pxls	%myelin in NAWM	Average %myelin NAWM
NAWM region 1	57754	58799	98,2	
NAWM region 2	33991	34819	97,6	97,8 %
NAWM region 3	16917	17362	97,4	

Encephalitis ROI

Black Pxls ROI	Tot. Pxls	% Black Pxls in ROI
150284	215688	69,7 %

Threshold value set
T203

Myelin-% in encephalitis ROI vs. NAWM ROIs

Real myelin-% in encephalitis area
71,3 %

- - - = ROI surrounding the area affected by encephalitis
- - - = ROI surrounding blood vessel
- - - = ROI of 3 different NAWM areas

Figure S3. Myelin quantification in an encephalitis brain section with ImageJ. **(A)** The percentage of myelin was quantified from images of LFB-stained sections, with myelin stained blue; **(B)** The images were processed into an 8-bit binarized mask of myelin, with the black pixels representing the myelin. A threshold value was set at a level that most closely matched the true image (in LFB), in order to retain as many true myelin pixels as possible. The ratio of black pixels was obtained by dividing the number of black pixels by the total amount of pixels in each defined region of interest (ROI). The total pixel amount and black pixels of a blood vessel (ROI surrounding blood vessel, present within the area affected by encephalitis) were subtracted from the total pixel amount and black pixel amount of the ROI surrounding the area affected by encephalitis, to obtain only percentages of myelin. The ROIs in the NAWM were drawn as pictured above, excluding (large) blood vessels. The computed percentage of black pixels in the ROI of all 3 NAWM regions was averaged to a percentage of myelin. The number of black pixels then indicated the percentage of myelin in lesions, with 100% myelin being defined by the number of black pixels in NAWM (as NAWM represents normal myelin density). In this figure all ROIs are captured in one image. In reality, during data acquisition, images of each ROI (3 NAWM and 1 pathological area) were all taken separately at the same magnification using Phenochart (version 1.0.12, Perkin Elmer AKOYA Biosciences); The myelin quantification is calculated in the tables below, with 71.30% myelin present within the affected area as result.

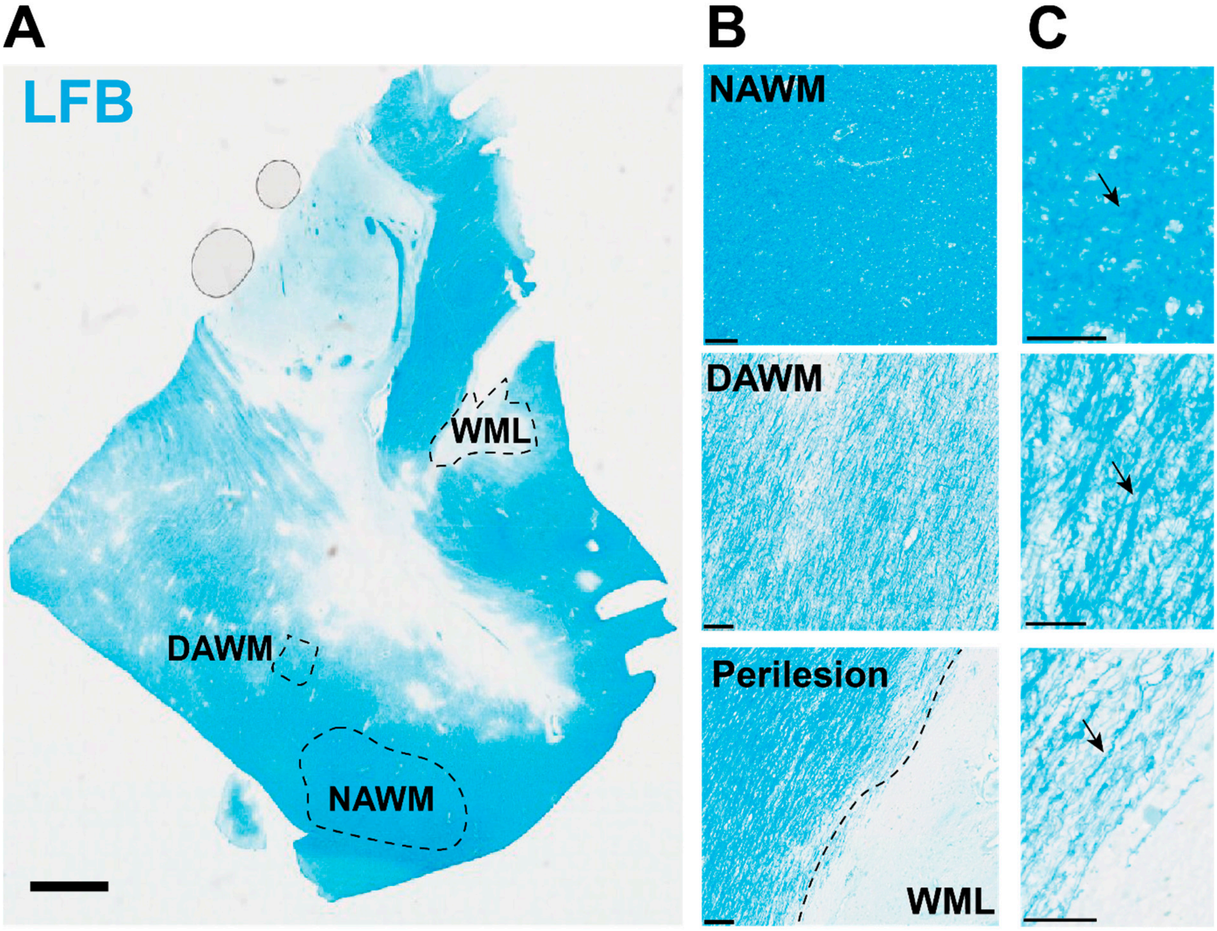

Figure S4. LFB detection of different WM regions in MS brain. **(A)** overview of a brain section from a donor with MS stained using LFB. **(B)** Images show a representative LFB staining in a region of NAWM, DAWM, and WML. Arrows in **(C)** indicate the presence of myelinated fibers in the different regions of MS white matter. Scale bars are 2 mm in **(A)**, 100 µm in **(B)** and 50 µm in **(C)**.
